# Supplementary material for: Clustering Genes of Common Evolutionary History
Source: Mol Biol Evol. 2016 Feb 17;33(6):1590–605. doi: 10.1093/molbev/msw038 (PMC4868114; doi:10.1093/molbev/msw038)
Supplement: Supplementary Data [file supp_33_6_1590__index.html]

Clustering Genes of Common Evolutionary History — Clustering Genes of Common Evolutionary History — Supplementary Data 

# Clustering Genes of Common Evolutionary History

## Supplementary Data

files

- Supplementary Data - pdf file
