# Supplementary material for: Clustering Genes of Common Evolutionary History
Source: Mol Biol Evol. 2016 Feb 17;33(6):1590–605. doi: 10.1093/molbev/msw038 (PMC4868114; doi:10.1093/molbev/msw038)
Supplement: Supplementary Data [file supp_msw038_Supplementary_Materials.pdf]

Supplementary Figures and Tables

Clustering genes of common evolutionary history

Gori et al.

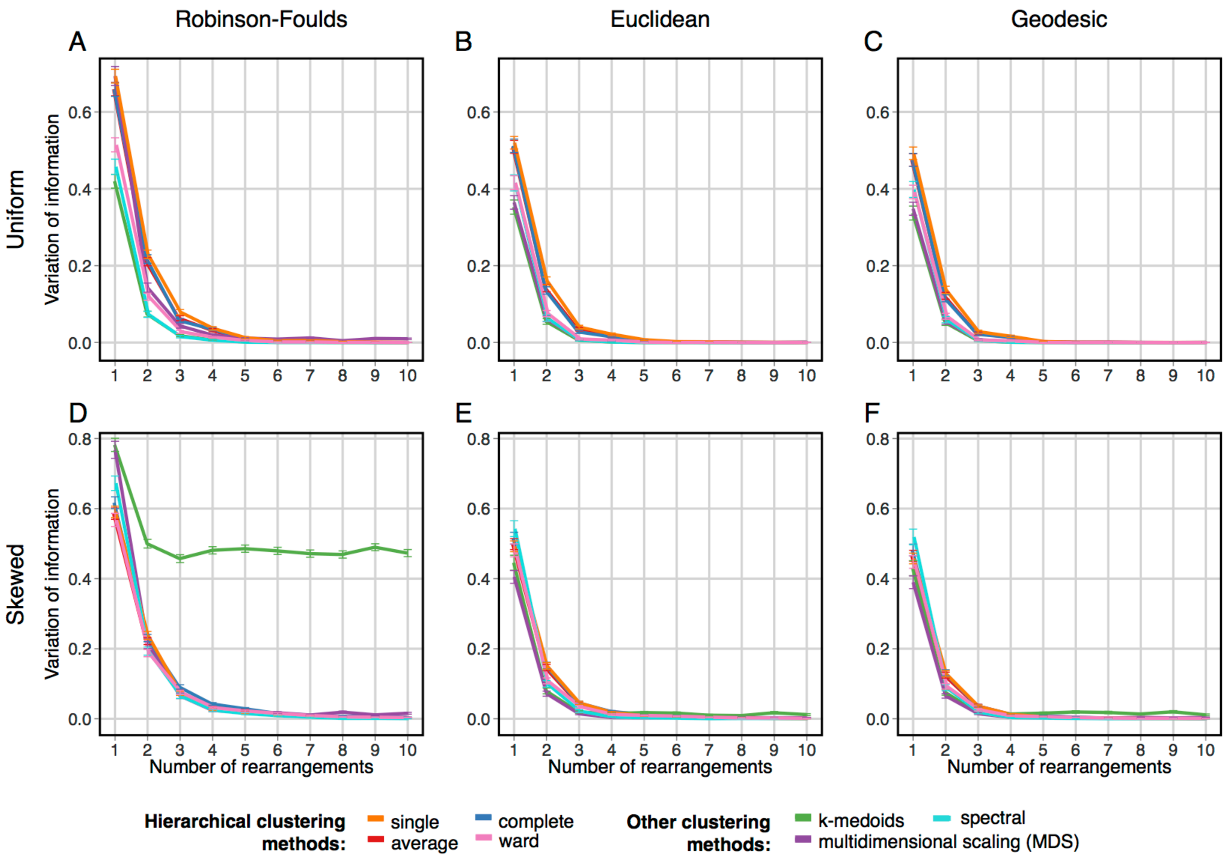

Supplementary Figure 1: ***Small data set, SPR rearrangements.*** *Panels show the relative performances of combinations of distance metric (varying over columns of panels) and clustering methods (shown by the colours of the lines), as measured by the variation of information metric (y-axes), which is a measure obtained when comparing the inferred solution with the true solution (higher values show a larger departure from the correct solution). Lines show the mean value obtained from 1000 replicates, and the error bars show the standard error of the mean. Rows correspond to the experiments with a partition of uniformly-sized clusters (A–C) and those with a partition of clusters of skewed sizes (D–F). In each individual panel, the x-axis represents the number of SPR rearrangements separating the underlying clusters, so that increasing values along this axis correlate with the clustering problem becoming easier.*

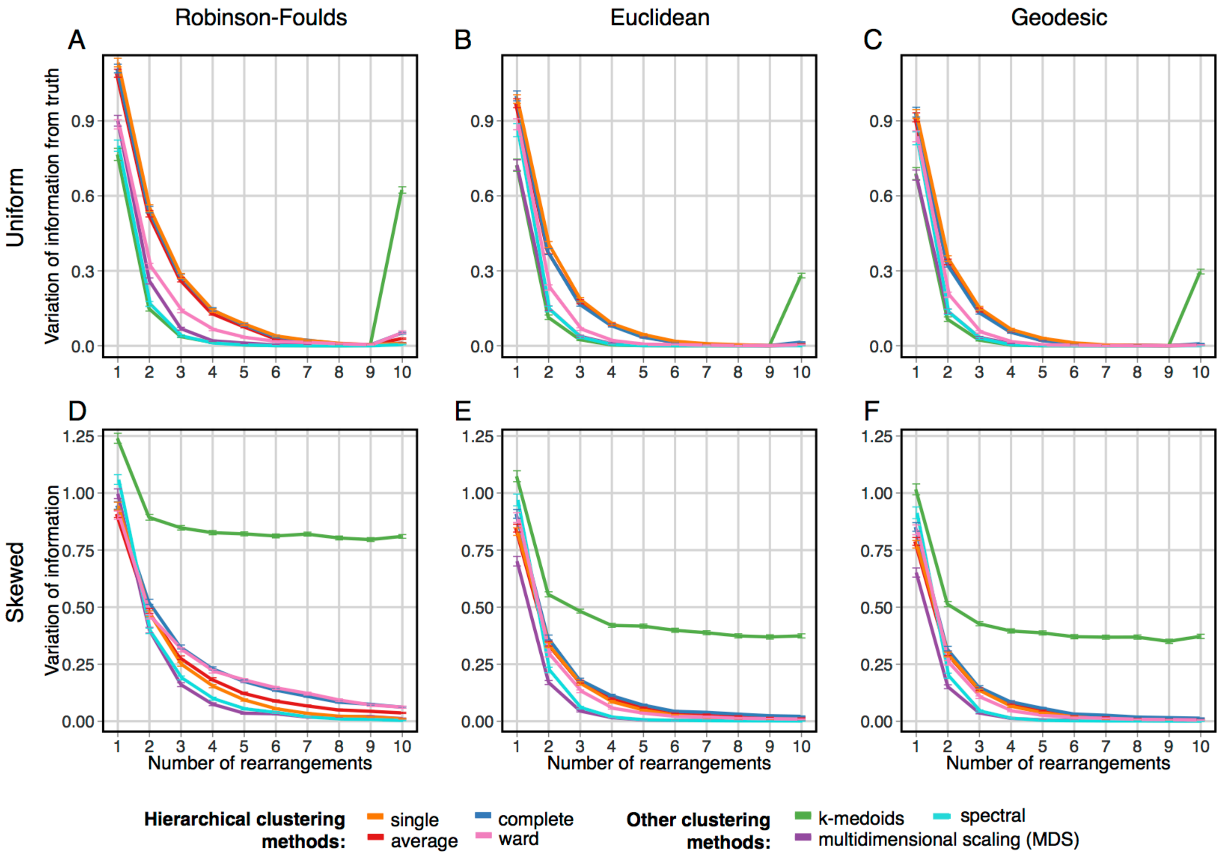

Supplementary Figure 2: ***Large data set, SPR rearrangements.*** *Panels show the relative performances of combinations of distance metric (varying over columns of panels) and clustering methods (shown by the colours of the lines), as measured by the variation of information metric (y-axes), which is a measure obtained when comparing the inferred solution with the true solution (higher values show a larger departure from the correct solution). In each individual panel, the x-axis represents the number of SPR rearrangements separating the underlying clusters.*

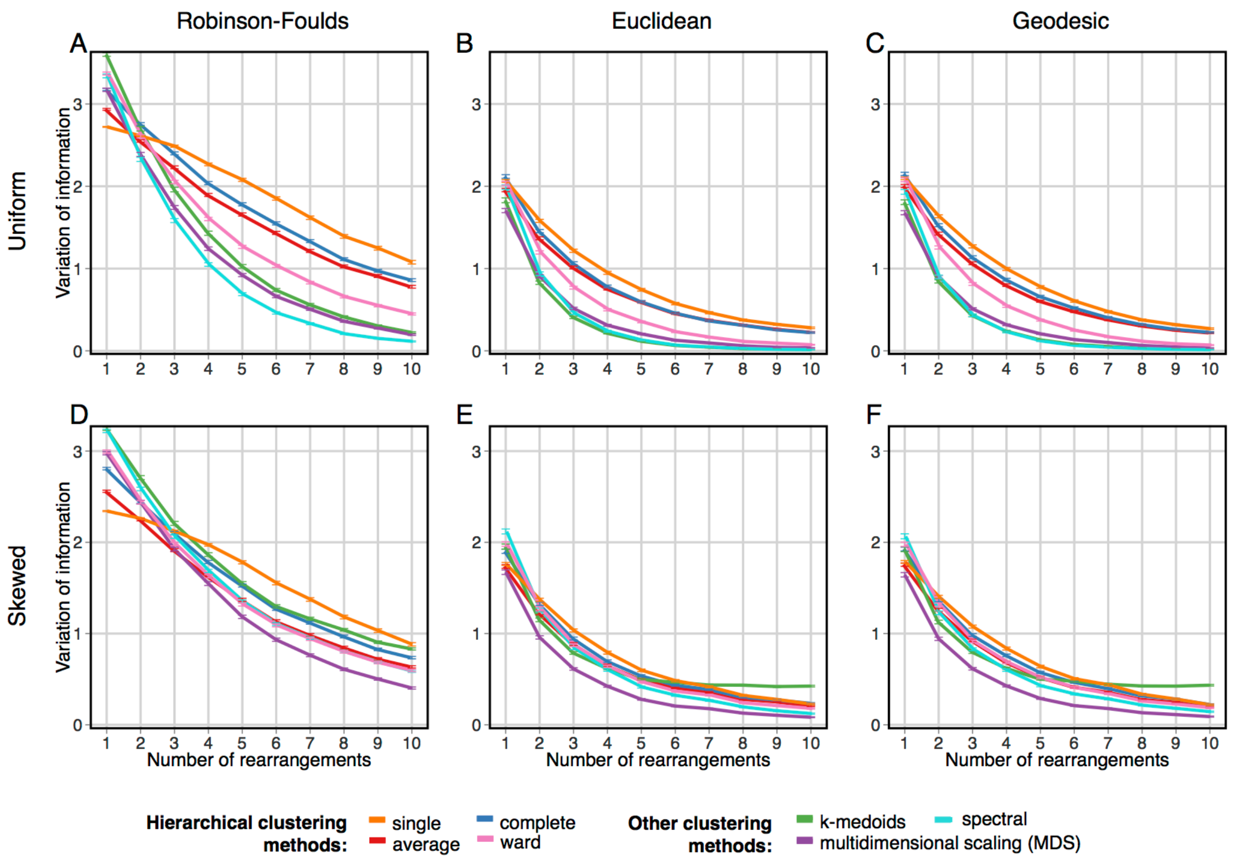

Supplementary Figure 3: ***Large data set, NNI rearrangements.*** *Panels show the relative performances of combinations of distance metric (varying over columns of panels) and clustering methods (shown by the colours of the lines), as measured by the variation of information metric (y-axes), which is a measure obtained when comparing the inferred solution with the true solution (higher values show a larger departure from the correct solution). In each individual panel, the x-axis represents the number of NNI rearrangements between underlying clusters.*

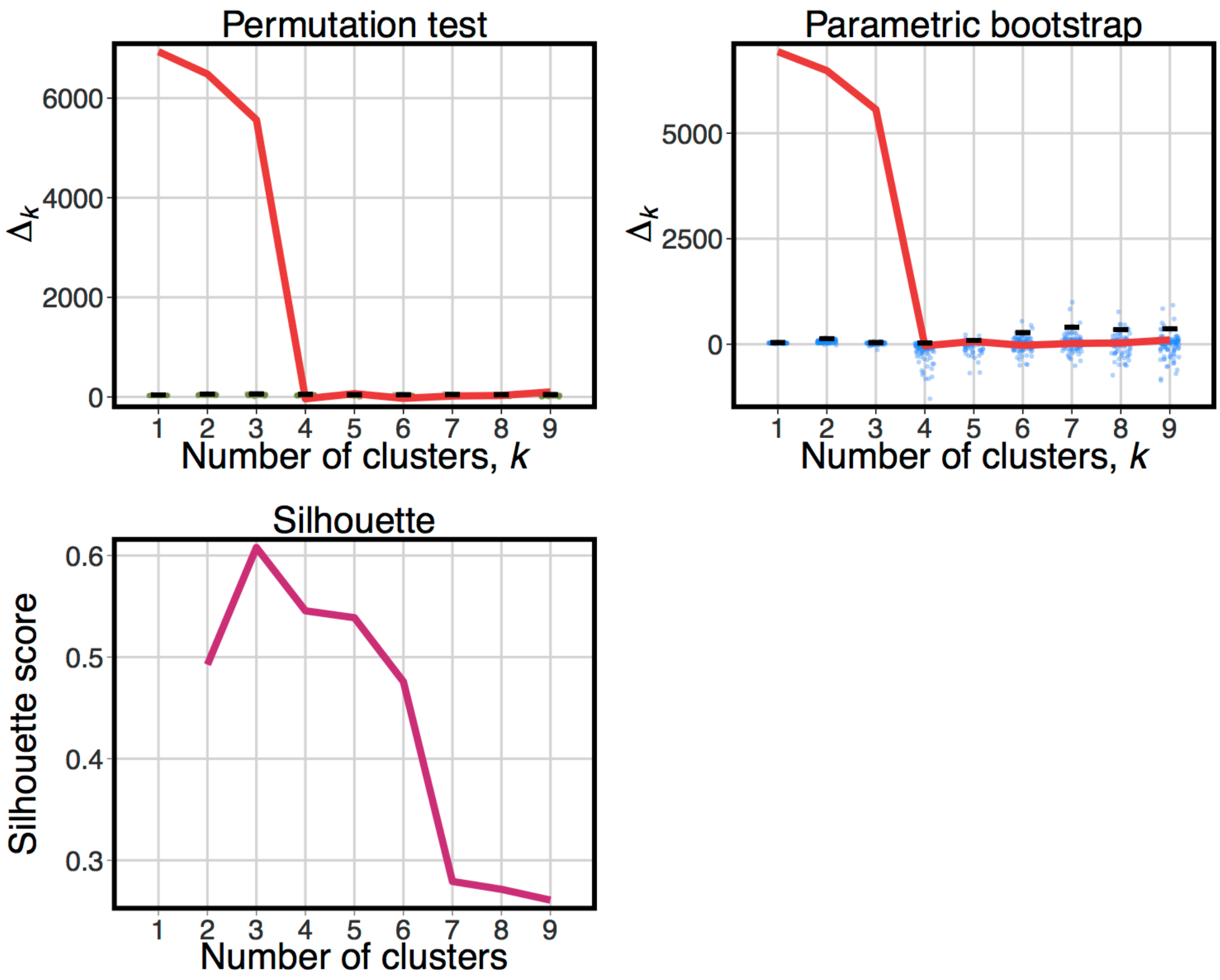

Supplementary Figure 4: Comparison of the criteria used to determine the number of clusters on a single problem instance with true number of clusters equal to four. The simulation scenario is the same as in Figure 3, however this figure shows an instance for which the criteria do not all agree on the number of clusters. (A) Permutation test: the improvement in likelihood for each additional cluster (red curve) is significantly greater than that observed for permuted data sets (green dots show the distribution of values over 100 permutations) until the comparison between 4 and 5 clusters is reached, correctly implying that the use of 4 clusters is optimal. (B) Parametric bootstrap test: again, the improvement for each additional cluster (red curve) is significantly greater than that for data sets simulated for one fewer cluster (blue dots) until the true number of clusters (4) has been reached. (C) the silhouette score, a general-purpose stopping criterion, has its maximum at a value of 3. In this instance, the newly devised methods give the correct answer, while the general purpose silhouette criterion underestimates the number of clusters.

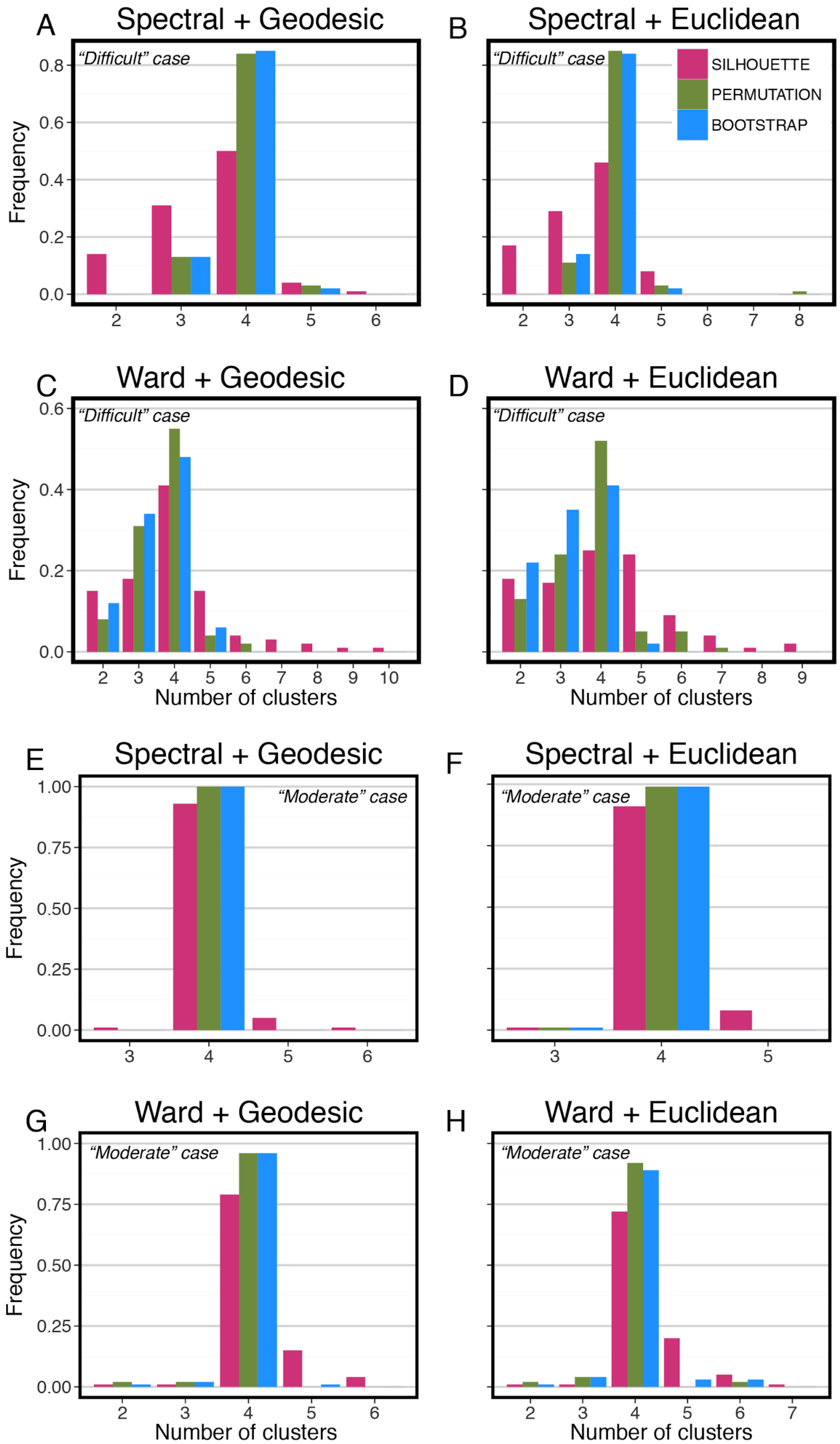

Supplementary Figure 5: Distributions of the number of clusters found for 100 "difficult" problem instances, analysed under the four combinations of spectral / Ward's method clustering and Euclidean / geodesic distances. In every instance the true number of clusters is 4. In each separate case the special-purpose permutation and bootstrap methods outperform the general-purpose silhouette method at selecting the correct number of clusters. When wrong, all methods tend towards underestimation rather than overestimation. Spectral clustering outperforms Ward's method clustering, and geodesic distances slightly outperform Euclidean.

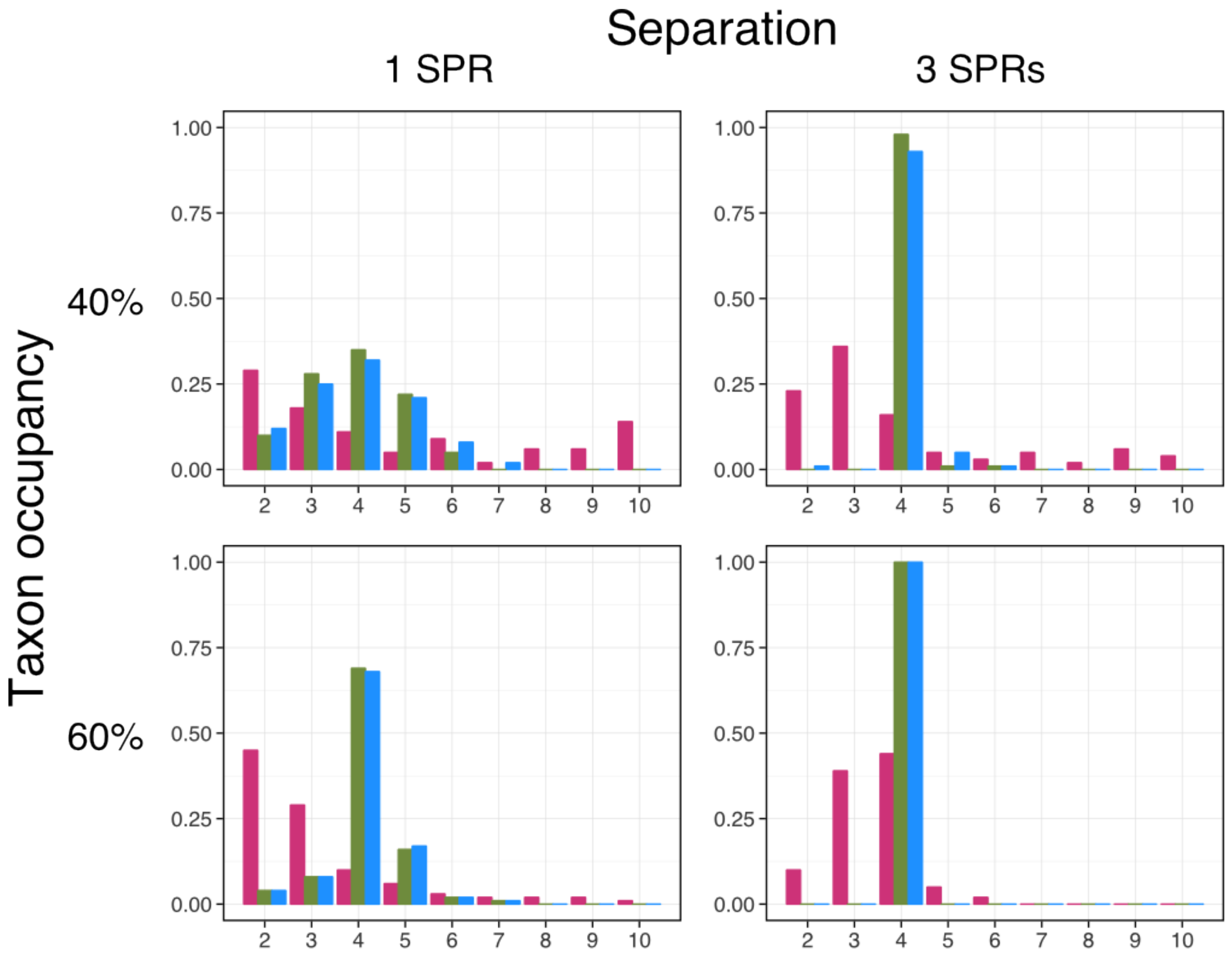

Supplementary Figure 6: The performance of criteria for determining the number of clusters, with sparse data. Four examples are shown, for two levels of occupancy (40 and 60%; rows), and two levels of cluster separation (1 and 3 SPRs; columns). Occupancy is expressed as a percentage; 40%, for example, means that each taxon was included in any particular locus with probability 0.4.

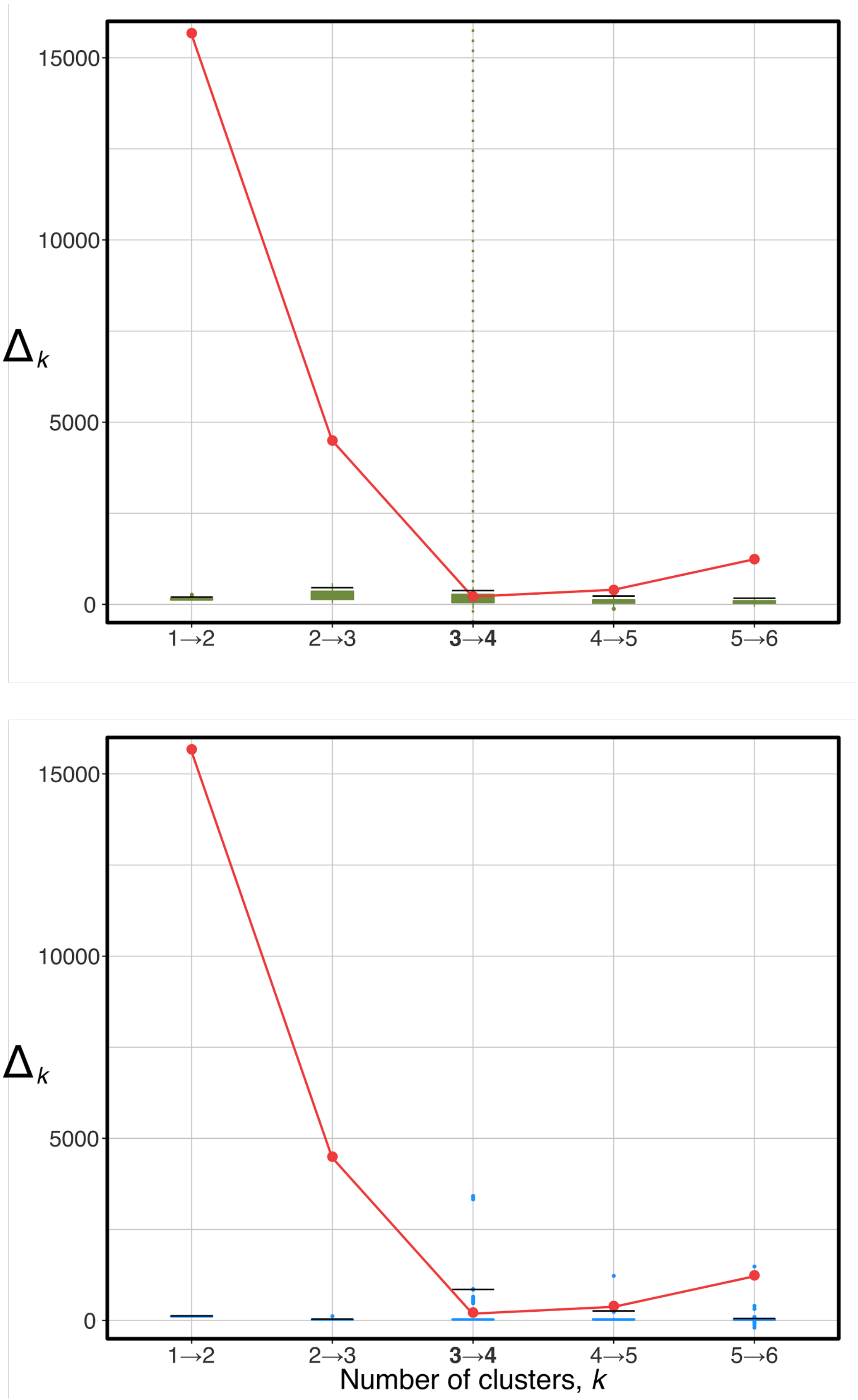

Supplementary Figure 7: Stopping criteria applied to yeast dataset. The top-most panel shows the result of applying the permutation-based (non-parametric) variant of the stopping criterion to the yeast dataset. The lower panel shows the result of applying the parametric bootstrap variant of the stopping criterion. Both variants suggest that the data should be partitioned into three clusters.

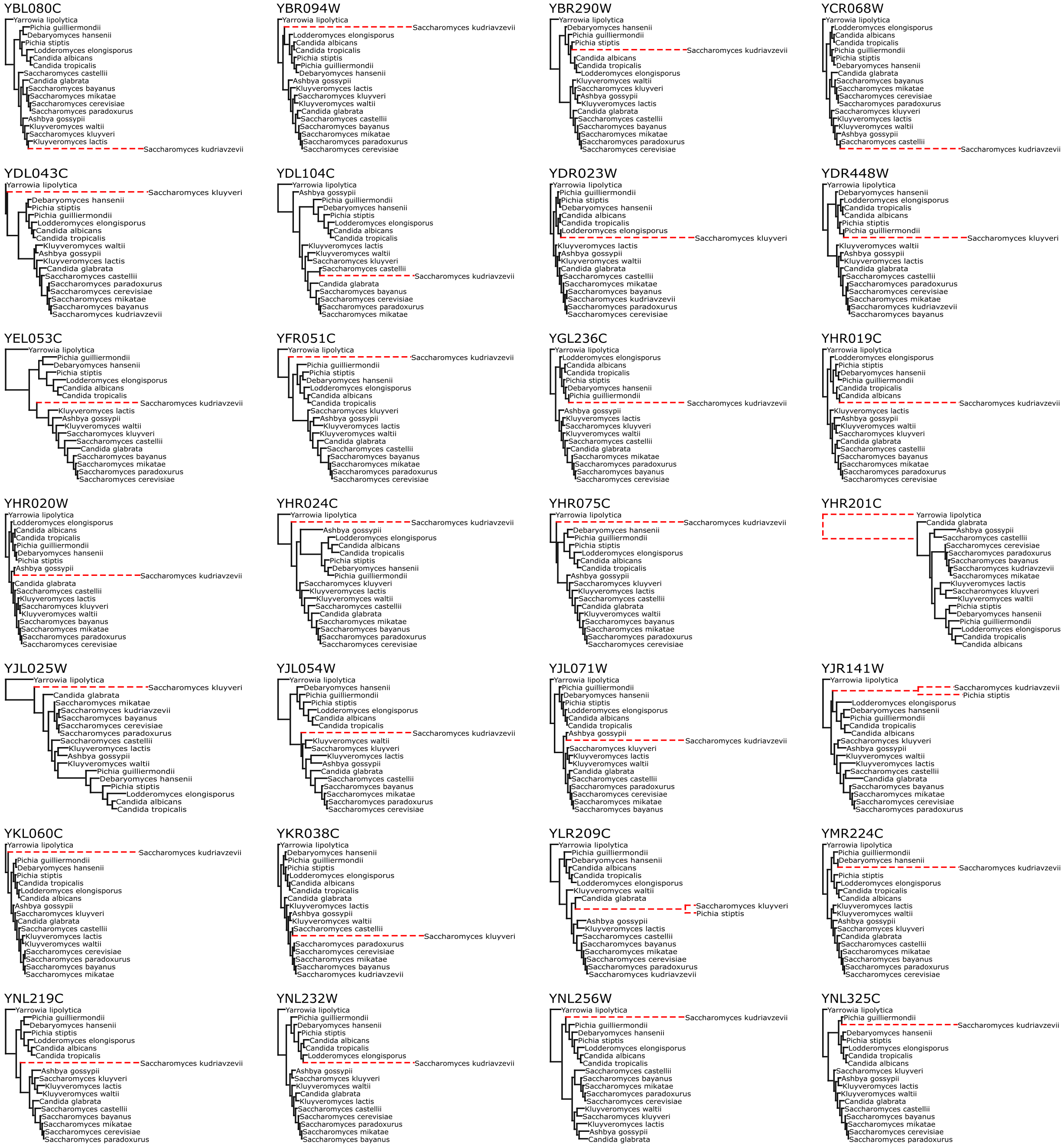

YBL080C
YBR094W
YBR290W
YCR068W
YDL043C
YDL104C
YDR023W
YDR448W
YEL053C
YFR051C
YGL236C
YHR019C
YHR020W
YHR024C
YHR075C
YHR201C
YJL025W
YJL054W
YJL071W
YJR141W
YKL060C
YKR038C
YLR209C
YMR224C
YNL219C
YNL232W
YNL256W
YNL325C

(*continued...*)

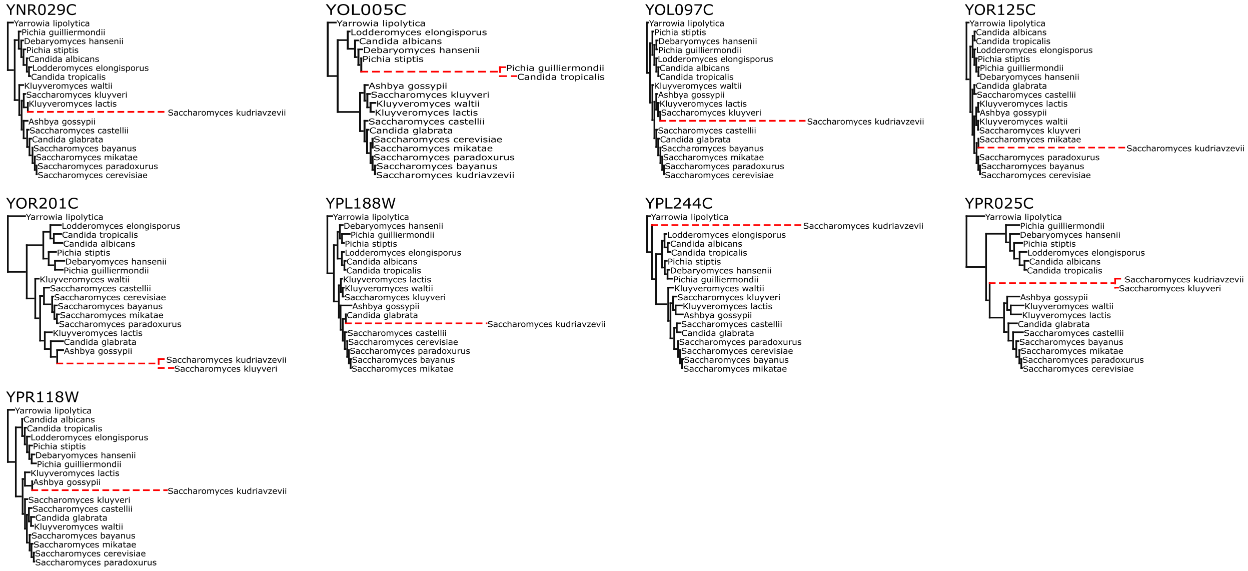

Supplementary Figure 8: Phylogenetic trees for the 37 yeast loci discovered to have erroneous orthology, with the non-orthologous sequences highlighted as red dashed lines.

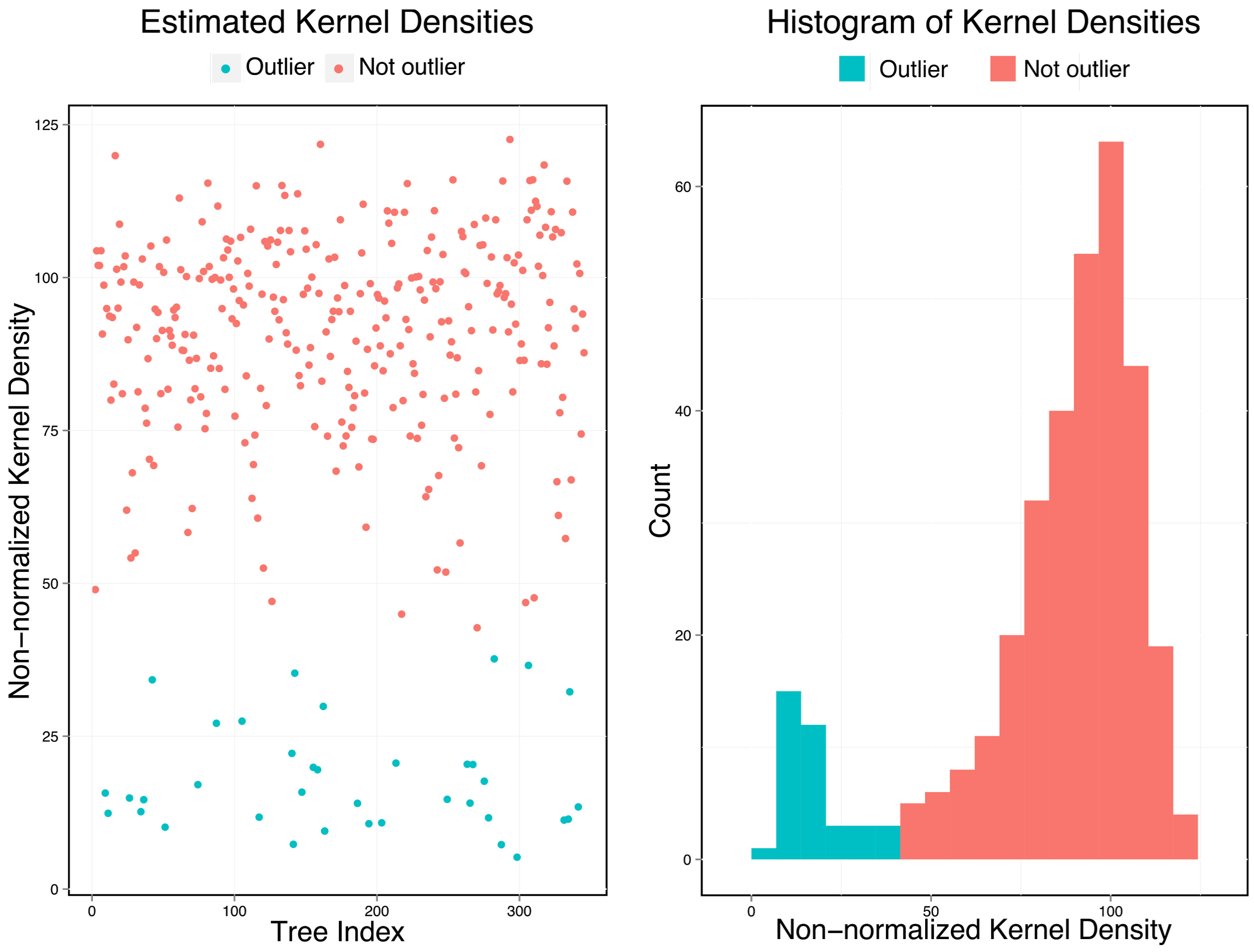

Supplementary Figure 9: Application of kdetrees to the yeast dataset. The scatterplot in the left panel shows the kernel density estimate for each tree. The order of the trees along the x-axis is arbitrary with respect to cluster membership, rather being derived from the alphabetical ordering of the names of the loci. The right panel shows a histogram of the kernel density scores. In both panels, the outliers are coloured blue.

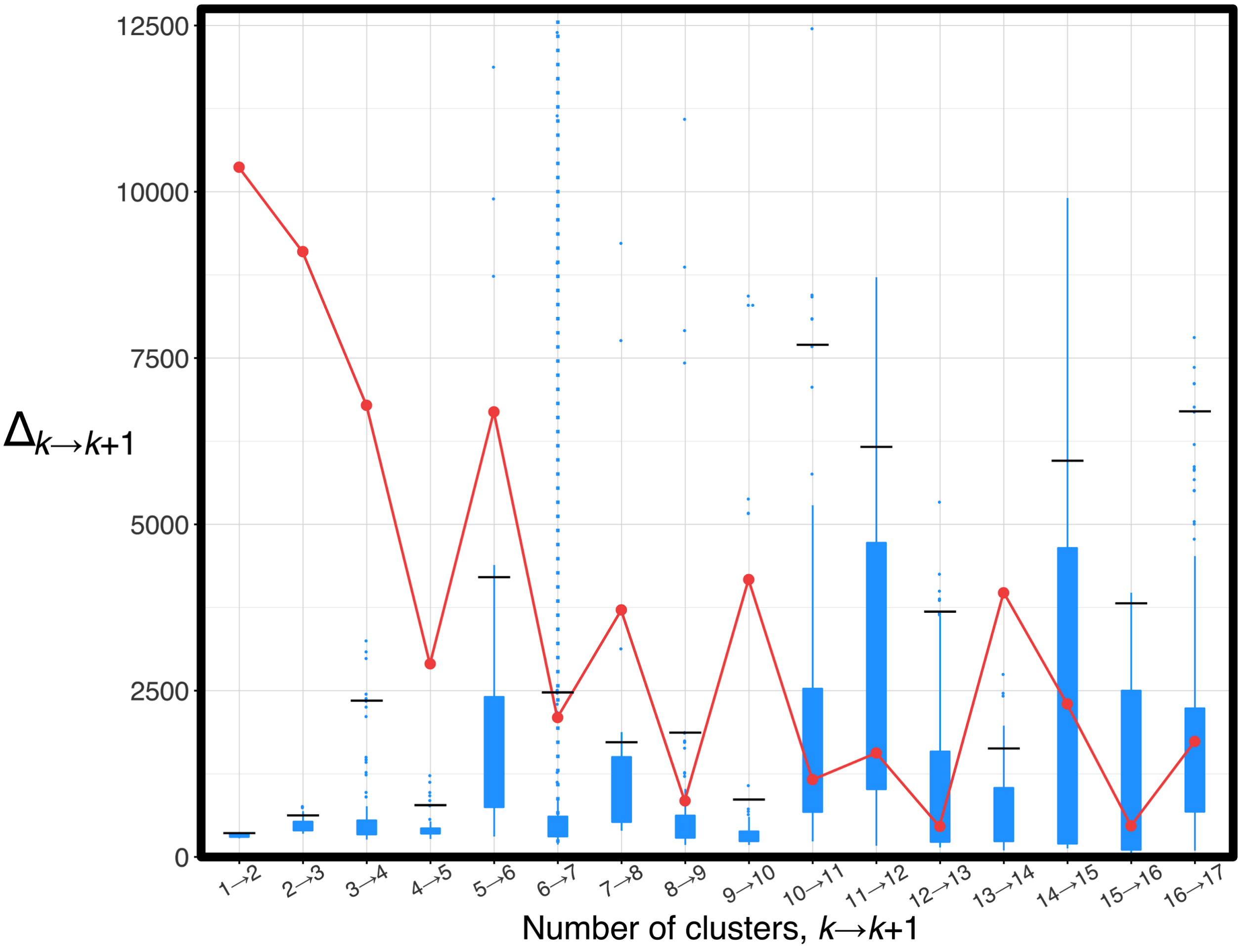

Supplementary Figure 10: Likelihood improvement gained when partitioning the Chiastocheta *data into increasing numbers of clusters (red points), using the parametric bootstrap criterion. The number of clusters selected by the stopping criterion is indicated by the vertical dashed line. Compared with the permutation test (Figure 8), the parametric bootstrap procedure yields a much larger variance in the likelihood improvement. Examination of the data reveals that this is due to the shallowness of some cluster trees, which makes it challenging for treeCl to identify the optimal clusters under both the null and alternative hypotheses. However, the conclusion (at least 4 clusters) is consistent with that of the permutation test.*

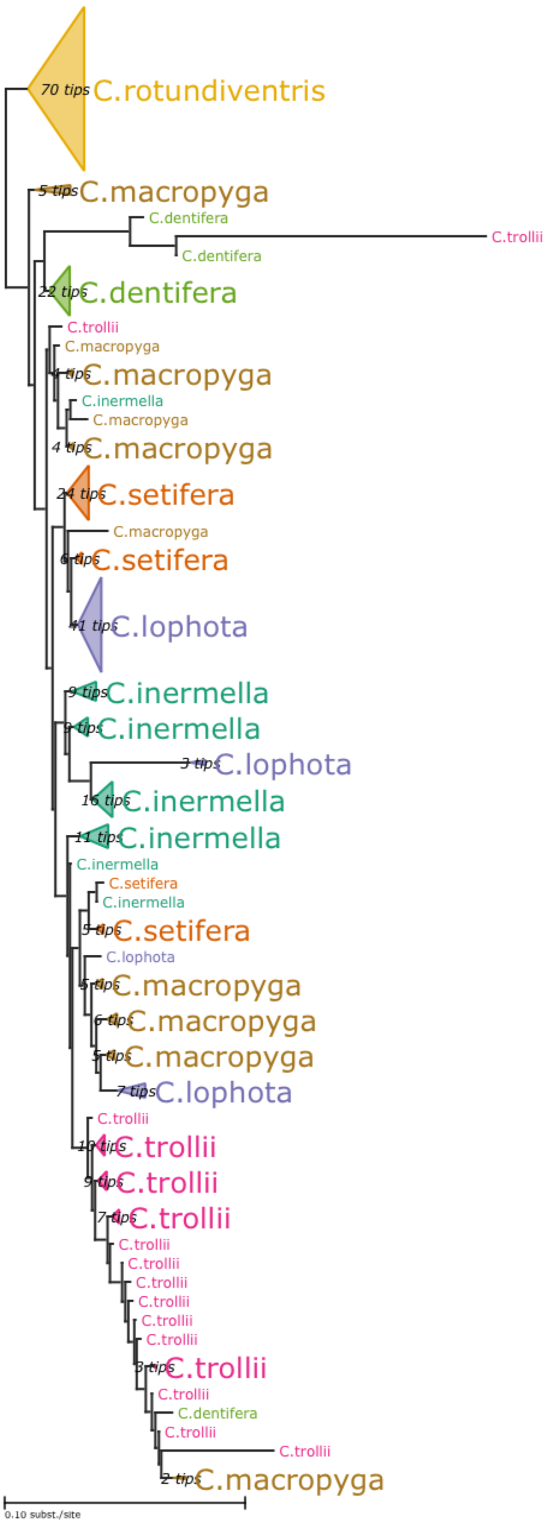

Supplementary Figure 11: A tree obtained from one of the clusters when partitioning Chiastocheta *loci into 5 clusters. The species are no longer monophyletic.*

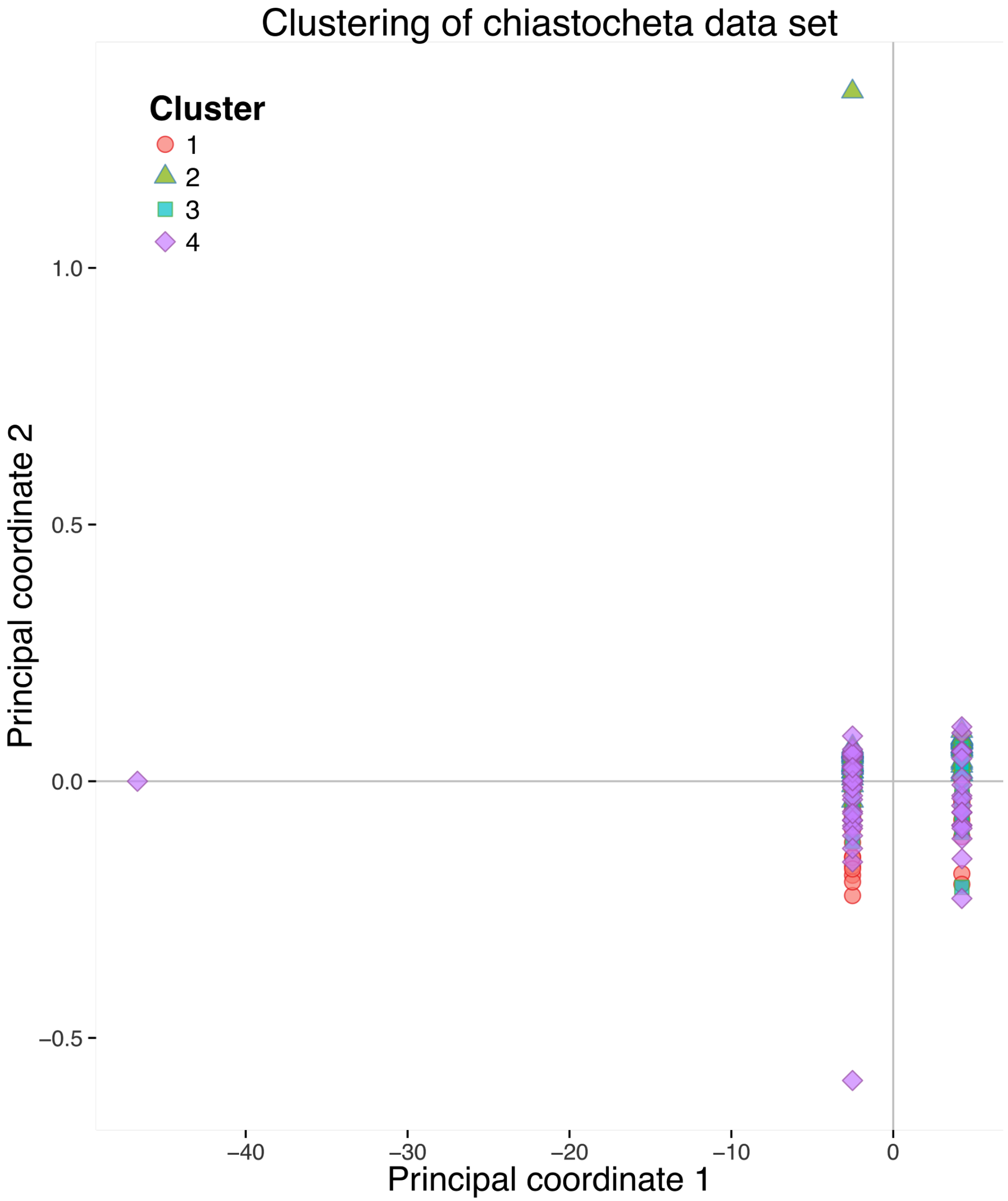

Supplementary Figure 12: Embedding of chiastocheta trees using classical multidimensional scaling. With the exception of three outliers, the trees form two groups that are clearly separated by the first principal coordinate. However, this separation is not indicative of the cluster structure detected by treeCl using spectral clustering. Classical multidimensional scaling can be distorted when the input distances are not Euclidean (Torgerson 1952; Gower and Legendre 1986). In this case, non-Euclidean distances may arise from missing species' sequences for some loci potentially causing violations of the triangle equality amongst the inter-locus tree distances.

**Orthologous Group**	**Misannotated Species**	**Best hit in S. cerevisiae**
YBL080C	*Saccharomyces kudriavzevii*	YMR219W
YBR094W	*Saccharomyces kudriavzevii*	YLR357W
YBR290W	*Saccharomyces kudriavzevii*	YLR114C
YCR068W	*Saccharomyces kudriavzevii*	YJR107W
YDL043C	*Saccharomyces kluyveri*	YDL051W
YDL104C	*Saccharomyces kudriavzevii*	YKR038C
YDR023W	*Saccharomyces kluyveri*	YHR011W
YDR448W	*Saccharomyces kluyveri*	YFR037C
YEL053C	*Saccharomyces kudriavzevii*	YOL080C
YFR051C	*Saccharomyces kudriavzevii*	YPL259C
YGL236C	*Saccharomyces kudriavzevii*	YBL098W
YHR019C	*Saccharomyces kudriavzevii*	YCR024C
YHR020W	*Saccharomyces kudriavzevii*	YER087W
YHR024C	*Saccharomyces kudriavzevii*	YLR163C
YHR075C	*Saccharomyces kudriavzevii*	YLR133W
YHR201C	*Yarrowia lipolytica*	YMR052C-A
YJL025W	*Saccharomyces kluyveri*	YDR285W
YJL054W	*Saccharomyces kudriavzevii*	YBL052C
YJL071W	*Saccharomyces kudriavzevii*	YPR185W
YJR141W	*Saccharomyces kudriavzevii*	YLR019W
	Pichia stipitis	YNL144C-like
YKL060C	*Saccharomyces kudriavzevii*	YER043C
YKR038C	*Saccharomyces kluyveri*	YDL104C

YLR209C	*Saccharomyces kluyveri*	YLR017W
	Pichia stipitis	YLR017W
YMR224C	*Saccharomyces kudriavzevii*	YAL035W
YNL219C	*Saccharomyces kudriavzevii*	YGL142C
YNL232W	*Saccharomyces kudriavzevii*	YBL052C
YNL256W	*Saccharomyces kudriavzevii*	YPL070W
YNL325C	*Saccharomyces kudriavzevii*	YNL106C
YNR029C	*Saccharomyces kudriavzevii*	YPL009C
YOL005C	*Candida tropicalis*	YNL113W
	Pichia guilliermondii	YNL113W
YOL097C	*Saccharomyces kudriavzevii*	YGR185C
YOR125C	*Saccharomyces kudriavzevii*	YER086W
YOR201C	*Saccharomyces kudriavzevii*	YLR051C
	Saccharomyces kluyveri	YLR051C
YPL188W	*Saccharomyces kudriavzevii*	YKR056W
YPL244C	*Saccharomyces kudriavzevii*	YEL004W
YPR025C	*Saccharomyces kudriavzevii*	YNL025C
	Saccharomyces kluyveri	YNL025C
YPR118W	*Saccharomyces kudriavzevii*	YKR026C

Supplementary Table 1: Summary of erroneous orthology discovered in the yeast data set. The first column gives the orthologous group to which the sequences from the species in the second column were assigned. The third column gives the gene name of the best BLAST hit in S. cerevisiae*.*
